# Therapeutic effects of chlorogenic acid on allergic rhinitis through TLR4/MAPK/NF-κB pathway modulation

**DOI:** 10.17305/bb.2024.11582

**Published:** 2024-12-30

**Authors:** Xiaoyan Xu, Lei Wang, Guangyao Wu, Xixia Li

**Affiliations:** 1 Otolaryngology Head and Neck Surgery, China Resources & Wisco General Hospital, Wuhan, Hubei, China

**Keywords:** Chlorogenic acid, CGA, RAW264.7 cells, allergic rhinitis, AR, TLR4/MAPK/NF-κB pathway, inflammation

## Abstract

Chlorogenic acid (CGA) exhibits promising anti-inflammatory properties, making it a potential therapeutic agent for inflammatory conditions and allergic rhinitis (AR). This study aimed to evaluate the therapeutic effects of CGA on inflammation in RAW264.7 macrophage cells and on AR in mice. RAW264.7 cells were treated with lipopolysaccharide (LPS) to induce inflammation and cultured with varying concentrations of CGA, a *Tlr4*-silenced gene (*shTlr4*) transfection, and the MAPK/NF-κB pathway activator diprovocim. Cell viability was assessed using the CCK8 assay, while levels of nitric oxide (NO), TNF-α, and IL-6 were measured by Griess colorimetry, immunofluorescence, and ELISA. Expression and phosphorylation levels of the MAPK/NF-κB pathway were evaluated using qPCR and western blotting. Additionally, ovalbumin (OVA)-induced AR mice received different doses of CGA, and Toll-like receptor-4 (*Tlr4*) overexpression was induced. *In vitro*, CGA treatment significantly reduced LPS-induced cell activity, NO, TNF-α, and IL-6 secretion, and downregulated *Tlr4*, p-p38, p-p65, and p-IκB expression. *Tlr4* inhibition suppressed cell activity and inflammation by blocking MAPK/NF-κB pathway activation. Conversely, *Tlr4* overexpression counteracted the effects of CGA, increasing cell activity and inflammatory factor concentration. In OVA-induced AR mice, CGA effectively alleviated allergic symptoms, reduced inflammatory factor secretion, and inhibited TLR4/MAPK/NF-κB pathway activity. These findings suggest CGA’s potential as an anti-inflammatory agent in RAW264.7 cells and AR models through modulation of the TLR4/MAPK/NF-κB pathway.

## Introduction

Allergic rhinitis (AR) is a Th2-type immunoreactive disease of the nasal mucosa, primarily mediated by IgE, which occurs when atopic individuals are exposed to allergens under the combined influence of genetic and environmental factors [1]. Typical AR symptoms include paroxysmal sneezing, excessive nasal discharge, nasal congestion, and nasal itching. Severe cases may also involve headaches, dizziness, and tinnitus [2]. Research indicates that AR is often accompanied by other diseases, has a high global incidence, and poses a significant burden on patients’ quality of life [3]. Despite its prevalence, the etiology of AR remains poorly understood due to the diversity of pathogenic factors. As a result, current treatments mainly focus on symptom control rather than targeting the underlying mechanisms, highlighting the need for effective therapeutic interventions [4]. Understanding the pathogenesis of AR and identifying specific therapeutic targets remain critical areas of research. From an immunological perspective, the essence of AR and related allergic diseases lies in the Th2-cell-mediated inflammatory response involving eosinophils (Eos) [5]. Recent studies have found that Th2 cytokines, such as IL-4 and IL-13, can induce the polarization of macrophages into the M2 phenotype via STAT6 activation. M2 macrophages, in turn, produce cytokines and chemokines—including arginine-1 (Arg-1), CCL17, and CCL24—that regulate Eos and mediate allergic inflammatory reactions [6]. Toll-like receptor-4 (TLR4), a key regulator of immune responses, is widely expressed on inflammatory cells and lymphocytes [7]. Evidence shows that TLR4 can either exacerbate or mitigate allergic airway diseases. For instance, TLR4 protein expression in the nasal mucosa increases following allergen exposure in AR patients during pollen seasons [8, 9]. Furthermore, inhibiting TLR4 signaling pathways can modulate macrophage-driven inflammation, offering potential therapeutic benefits for AR [10]. Animal studies have demonstrated that siRNA-mediated downregulation of TLR4 in AR models reduces eosinophil numbers, inhibits Th2-related cytokine secretion, and improves AR symptoms [11]. Thus, targeting TLR4-mediated inflammatory responses could be a promising strategy for alleviating AR. Current AR treatments include drug therapy and immunotherapy. Drug therapies—comprising antihistamines, glucocorticoids, and antileukotriene drugs—provide rapid symptom relief but often have unstable long-term efficacy and can cause significant side effects [12–14]. This underscores the need to explore new drugs and therapeutic strategies. One potential candidate is chlorogenic acid (CGA), a bioactive compound derived from honeysuckle with the chemical formula C_16_H_18_O_9_ [15]. CGA exhibits antibacterial, anti-inflammatory, and antiviral properties, in addition to cardiovascular protective effects via cholesterol reduction [16, 17]. Studies have shown that CGA can reduce serum IgE, ROR-γt, and IL-17A levels in AR mice, thereby alleviating AR symptoms [18]. Moreover, CGA has been found to regulate the Th1/Th2 immune balance, reducing the inflammatory response associated with AR [19]. These findings suggest that CGA holds therapeutic potential for AR, although its exact mechanism of action remains unclear. In summary, CGA has demonstrated the ability to alleviate AR symptoms by inhibiting inflammation through anti-inflammatory pathways. However, the regulatory mechanisms underlying CGA’s effects on AR-related inflammation are still not fully understood. We hypothesize that CGA may exert its therapeutic effects on AR by modulating the TLR4-mediated inflammatory response. To test this hypothesis, this study employed RAW264.7 cells to establish an *in vitro* inflammation model of AR and evaluate CGA’s inhibitory effects on the inflammatory response. Additionally, an AR mouse model was constructed to further investigate CGA’s impact on AR symptoms and inflammation.

## Materials and methods

### Experimental materials

CGA was obtained from Shanghai Yuanye Biotechnology Co., Ltd., and mouse RAW264.7 cells were sourced from the Cell Bank of the Chinese Academy of Sciences. Additional materials included DMEM complete medium (Zhongqiao Xinzhou Co., Ltd.), lipopolysaccharide (LPS) (Beijing Solaibao Technology Co., Ltd.), and mouse nitric oxide (NO) assay kits (Biyuntian, Cat. S0020S). Greiss reagent (Sigma, Cat. G4410) and ELISA kits for mouse IL-4 (Cat. H005-1-2), IL-6 (Cat. H007-1-2), and TNF-α (Cat. H052-1-2) were purchased from Nanjing Jiancheng BioEngineering Institute. Chicken OVA was supplied by Meilunbio Inc., while the mouse IgE and histamine kits were sourced from Shanghai Mori Xiong Technology Industrial Co., Ltd. Antibodies used included TLR4 (Bioss, Cat. bs-20594R), P38 (Bioss, Cat. bs-0637R), p-P38 (Bioss, Cat. bs-0636R), P65 (Bioss, Cat. bs-0465R), p-P65 (Abcam, Cat. ab76302), IκB (Abcam, Cat. ab32518), p-IκB (Abcam, Cat. ab92700), and HRP-conjugated goat anti-rabbit IgG (Abcam, Cat. ab205718). Other reagents included the CCK-8 assay reagent (Bausch BioEngineering), total RNA extraction kit, TB Green^®^ Premix Ex Taq™ II FAST qPCR mix (Takara), and hematoxylin staining solution kit (Wuhan Google Biotechnology Co., Ltd.). The MAPK pathway inhibitor (SB203580) and activator (Diprovocim) were obtained from Med Chem Express (USA), and Giemsa staining solution was provided by Noyoung Bio.

### Cell culture

Mouse RAW264.7 cells were seeded in DMEM and cultured at 37 ^∘^C with 5% CO_2_. When the cells reached approximately 80% confluence, they were subcultured. To subculture, the adherent cells were detached using a pipette after adding complete medium. A 1 mL aliquot of the resulting cell suspension was mixed with 4 mL of complete medium in a culture flask, which was then returned to the incubator. LPS was dissolved in PBS and diluted to a 1 µg/mL solution using DMEM, as described in the literature [20]. Similarly, CGA was dissolved in PBS, and solutions at various concentrations (12.5, 25, 50, 100, 200, and 400 µg/mL) were prepared in DMEM based on the experimental requirements. RAW264.7 cells were then exposed to medium containing either LPS or CGA at different concentrations and cultured for 24 h prior to subsequent testing.

### CCK8 detection

RAW264.7 cells were seeded into culture plates, and 100 µL of DMEM solutions containing various drugs were added to each group, followed by incubation for 24 h. Each experiment was conducted in triplicate. After discarding the culture medium, 10 µL of CCK-8 solution was added to each well and incubated for 4 h. A blank control group and a negative control group were also included, with procedures identical to those of the experimental groups. The absorbance of each group was measured at a wavelength of 450 nm using a microplate reader (ELx800, BioTek, USA), and the cell survival rate was subsequently calculated.

### Cell transfection

The TLR4 overexpression plasmid (over-TLR4) was obtained from Hanheng Biotechnology (Shanghai) Co., Ltd. According to the manufacturer’s protocol, over-TLR4 was transfected into macrophages to induce TLR4 overexpression. The pUNO1 empty plasmid vector (Invivogen, Shanghai, China) served as the control (over-NC). Based on the mouse TLR4 gene sequence, short hairpin TLR4 RNA (shTLR4) and a negative control (sh-NC) were designed and constructed by Hanheng Biotechnology. The shTLR4 target sequence was TAGAGGTAGTTCCTAATATTA (21 bp), and the sh-NC target sequence was CCTAAGGTTAAGTCGCCCTCG. Lentiviral packaging of shTLR4 was performed using the target sequence. RAW264.7 cells were cultured to 60%–70% confluency and transfected with the plasmids using Lipofectamine 2000 Reagent (Invitrogen) according to the manufacturer’s instructions. Post-transfection, qRT-PCR was conducted to verify TLR4 expression levels.

### ELISA

Samples were collected from each group to determine protein concentrations following the kit instructions. The cell culture medium was centrifuged, and the supernatant was collected as the sample for measurement. Sample wells, standard wells, and blank wells were prepared by adding 50 µL of diluted sample, diluted standard, and blank DMEM, respectively. Each well was incubated with 50 µL of diluted detection antibody for 1.5 h. After incubation, the ELISA plate was washed six times with 300 µL of wash solution. Subsequently, 100 µL of horseradish peroxidase-labeled streptavidin was added and incubated for 30 min. The plate was washed again, followed by the addition of 100 µL of the chromogenic substrate TMB and incubation in the dark at 37 ^∘^C for 5–30 min. When the solution turned blue, the reaction was stopped by adding 100 µL of termination solution. The OD at 450 nm was measured using a microplate reader, and the results were analyzed using a standard curve.

### Detection of NO level (Griess method)

RAW264.7 cells were cultured with CGA for 24 h, followed by treatment with tissue lysate. Afterward, the cells were centrifuged, and the supernatant was collected. Fifty microliters of Griess I and Griess II reagents were added to each group and thoroughly mixed. The wavelength of the microplate reader was set to 540 nm, and the absorbance values were measured to calculate the concentration of NO.

### QRT-PCR

The cells were washed twice with PBS, resuspended in the appropriate lysis buffer, and allowed to stand for 2 min. RNA was then extracted using an RNA Spin Column. Following this, a reverse-transcription reaction system was prepared to synthesize cDNA. The resulting cDNA was subsequently added to the real-time PCR reaction system for amplification. The mRNA levels of all target genes were quantified relative to the reference gene ACTB. Gene expression was analyzed using the 2^−ΔΔCT^ method. The primers used in the experiment are listed in Table 1.

**Table 1:** Primer sequence

**Gene**	**Primer sequence (5′-3′)**	
TLR4	Forward	GAGCAAACAGCAGAGGAAGA
	Reverse	CCAGGTGAGCTGTAGCATTTA
P38	Forward	GAAAGCAGGGACCTTCTCATAG
	Reverse	GTGCTCAGGACTCCATTTCTT
P65	Forward	CCGACTTGTTTGGGTGATCT
	Reverse	TCCGTCTCCAGGAGGTTAAT
IKB	Forward	CCACTCCATGTAGCTGTCATC
	Reverse	CACGTAGGCTCCGGTTTATT
ACTB	Forward	GAGGTATCCTGACCCTGAAGTA
	Reverse	CACACGCAGCTCATTGTAGA

TLR4: Toll-like receptor-4.

### Western blotting

Cultured cells or tissue samples were washed twice with precooled PBS buffer. For tissue samples, the samples were ground and homogenized prior to processing. A volume of 200 µL of lysis buffer was then added to the cells, and the lysates were centrifuged at 12,000 rpm at 4 ^∘^C for 20 min to extract cellular proteins. The total protein concentration was quantified using the BCA method. Proteins were separated using a 10% SDS-PAGE gel electrophoresis system, followed by wet transfer onto membranes. The membranes were blocked with 5% skim milk powder for 2 h at room temperature, then incubated overnight with a primary antibody solution (diluted 1:1,000). After washing with TBST, the membranes were incubated with a secondary IgG antibody (diluted 1:10,000) for 1 h at room temperature. Subsequently, the membranes were treated with ECL solution for signal detection. The membranes were exposed for 5 min in an exposure box and then rinsed twice. Finally, the protein bands on the membranes were imaged and analyzed using the ChemiDoc XRS+ imaging system.

### Immunofluorescence

Cell slides were pre-placed into 24-well plates, and cells were subsequently added to the culture wells to promote their growth on the slides. After treating the cells with drugs and culturing them for 24 h, the cells were fixed with 200 µL of 4% paraformaldehyde for 15 min. This was followed by permeabilization using 200 µL of 0.2% Triton X-100 for 20 min. The cells were then blocked with 200 µL of 5% BSA at room temperature. Next, the slides were incubated with the primary antibody, followed by the fluorescent secondary antibody (dilution 1:5,000). Finally, the cells were stained with DAPI solution for 10 min. The slides were sealed with an anti-fluorescence quenching sealing agent, and fluorescence staining of the cells was directly observed under a laser confocal microscope (Olympus, FV3000).

### Establishment of AR mouse model

Male C57BL/6 mice (*n* ═ 35), aged 6–8 weeks, were purchased from Jiangsu Jichi Yaokang Biotechnology Co., Ltd. This study was approved by the Ethics Committee of China Resources & Wisco General Hospital. The mice were housed under standard conditions and allowed to acclimate for one week prior to the experiment. To establish the AR mouse model, mice in the model group were intraperitoneally injected with 200 µL of OVA sensitization solution-I—prepared with OVA, Al(OH)_3_, and normal saline—on days 0, 7, and 14. From days 21 to 27, the mice were intranasally challenged once daily with 10 µL of OVA sensitization solution-II (800 µg of OVA dissolved in 0.9% normal saline) administered to each side of the nasal cavity. Mice in the normal control group received the same dose of normal saline instead of OVA. On the 28th day, blood samples were collected, and the mice were sacrificed to isolate the nasal mucosal tissue. The samples were promptly frozen and stored for subsequent analysis.

### Group allocation and drug administration to mice

Before surgery, 35 mice were randomly divided into seven groups: the control group, the model group, the 50 mg/kg CGA group, the 100 mg/kg CGA group, the 200 mg/kg CGA group, the 200 mg/kg CGA + over-NC group, and the 200 mg/kg CGA + over-TLR4 group. All groups, except for the control group, were subjected to stimulation with OVA. Treatments were administered 30 min before OVA solution was delivered via nasal drip once daily, from days 21 to 27 of modeling (seven treatments total). Mice in the control and model groups received an equal volume of distilled water intragastrically. In the CGA treatment groups, AR mice were administered 50, 100, or 200 mg/kg of CGA intragastrically [21, 22]. Meanwhile, in the 200 mg/kg CGA + over-NC and 200 mg/kg CGA + over-TLR4 groups, AR mice received 200 mg/kg CGA intragastrically along with a nasal application of 20 µL of over-NC or over-TLR4 plasmid solution (1 µg/µL) once daily for seven days.

### Behavioral observation of mice

The mice were observed for sneezing, nose scratching, and runny nose behaviors over a 10-minute period, with observations recorded daily after nasal drops were administered on the 21st day. Animal behavior scores were evaluated, and a score exceeding five was deemed indicative of successful model establishment [23].

### Analysis of serum indexes

After 24 h of the final dose stimulation, the mice were anesthetized to collect orbital venous blood samples. The blood samples were allowed to stand for 1 h before being centrifuged at 4 ^∘^C for 15 min. The upper serum layer was carefully collected and stored in a freezer at 20 ^∘^C. The concentrations of histamine, IgE, TNF-α, IL-4, IL-5, and IFN-γ in the mouse serum were measured using the respective ELISA kits, following the procedures described in the ELISA test section. To analyze eosinophil levels, Giemsa and Wright staining solutions were prepared and applied to 5 µL of mouse venous blood. The stained blood samples were then examined under an orthographic microscope to count the number of Eos.

### Tissue sample collection

Twenty-four hours after the final nasal stimulation, the mice in each group were anesthetized via intraperitoneal injection of 10% chloral hydrate. The chest cavity was opened to expose the heart, and normal saline was perfused through the left ventricle. The nasal mucosa on both sides of the mice was carefully dissected and divided into two portions. One portion was immediately placed in neutral paraformaldehyde at 4 ^∘^C, fixed for 8 h, and subsequently used to prepare paraffin-embedded sections of nasal mucosa tissue. The other portion was processed by adding it to a lysis solution, followed by grinding and homogenization. This sample was then used to extract protein and RNA for further testing.

### Hematoxylin-eosin staining

The paraffin-embedded sections were mounted on slides and incubated at 37 ^∘^C overnight. The slides were then dehydrated sequentially in xylene, absolute ethanol, and 75% ethanol, followed by rinsing with tap water. Next, the sections were treated sequentially with hematoxylin, an aqueous hydrochloric acid solution, and an ammonia aqueous solution, with washing steps in between. Afterward, the sections were dehydrated using a graded alcohol series and stained with eosin staining solution for 5 min. Finally, the sections were treated with absolute ethanol, cleared using xylene, and sealed with neutral gum. Tissue staining was subsequently observed under a microscope.

### Ethical statement

The experimental protocols were approved by Medical Ethics Committee of the Second People’s Hospital of Guangdong Province.

### Statistical analysis

The experimental data are presented as mean ± standard deviation (SD). Statistical analysis and data visualization were performed using GraphPad 10.0. For pairwise comparisons, the LSD method was applied when variance was homogeneous, while Tamhane’s T2 method was utilized in cases of heterogeneous variance. A *P* value of less than 0.05 (*P* < 0.05) was considered indicative of a statistically significant difference.

## Results

### Effect of CGA on inflammatory response of RAW264.7 cells

**Figure 1.**
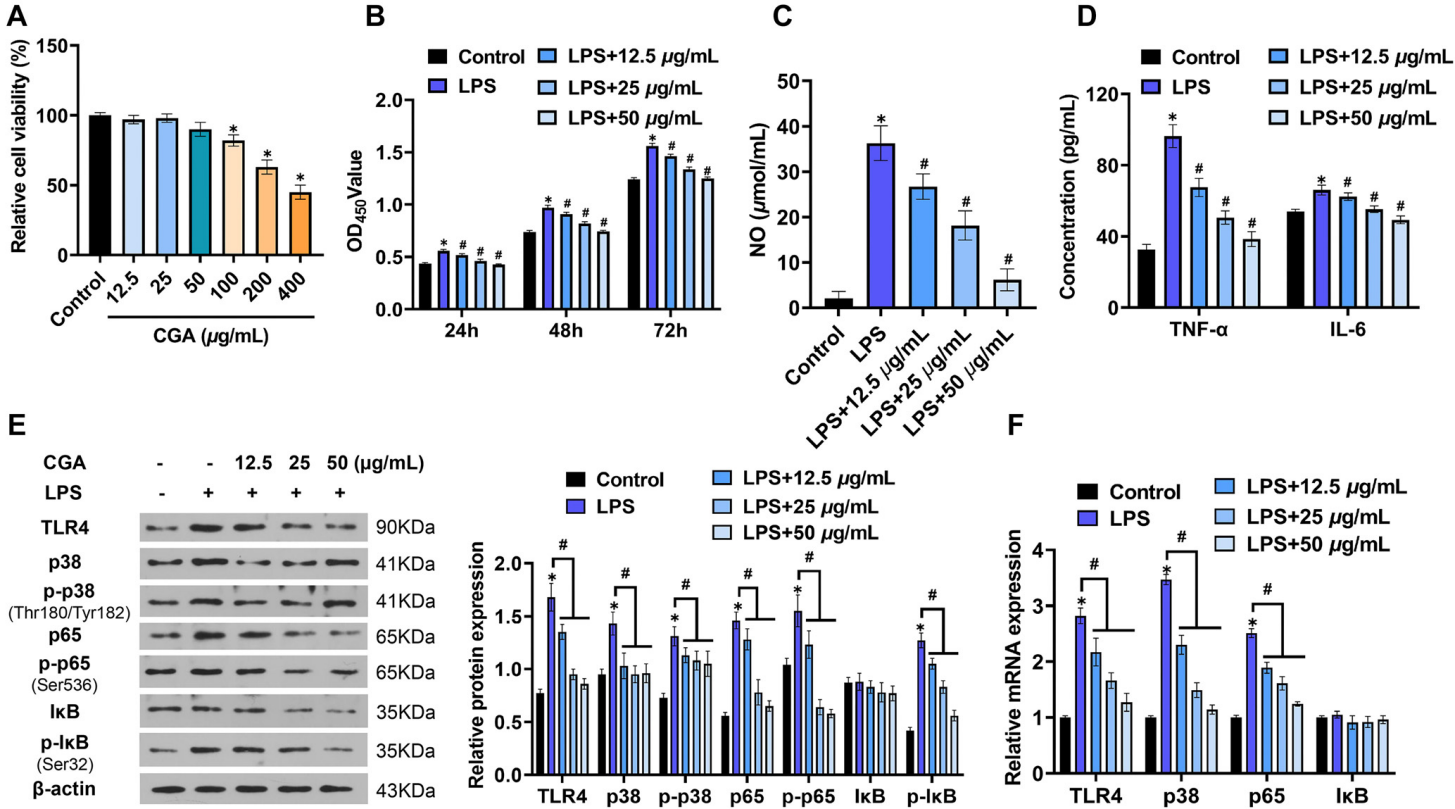
**Effect of culture with CGA on LPS-induced RAW264.7 cell function. ** (A) Cytotoxicity analysis of effects of CGA culture on cells; (B) Effects of CGA and LPS on cell proliferation; (C) The secretion of NO in cells was detected by the Griess method; (D) The secretion of TNF-α and IL-6 was detected by ELISA; (E) Effect of CGA on the protein expression of TLR4, p38, p-p38, p65, p-p65, IκB, and p-IκB in cells; (F) Effect of CGA on the mRNA expression of TLR4, p38, p65, and IκB in cells. *N* ═ 3, *P < 0.05, compared with control group; ^#^*P* < 0.05, compared with LPS group. LPS: Lipopolysaccharide; CGA: Chlorogenic acid; TLR4: Toll-like receptor-4.

First, the cytotoxic effect of CGA on cells was evaluated. Cell cultures treated with varying concentrations of CGA showed that, compared to the control group, CGA significantly inhibited cell viability at concentrations > greater than 50 µg/mL (*P* < 0.05). Consequently, three doses within the non-toxic concentration range were selected for subsequent experiments (Figure 1A). When cells were co-cultured with CGA and LPS for 48 h, LPS treatment alone enhanced cell viability compared to the control group. However, CGA at all three doses suppressed the LPS-induced increase in cell proliferation (Figure 1B). Additionally, CGA significantly inhibited the LPS-induced secretion of inflammatory factors, including NO, TNF-α, and IL-6, with the strongest inhibitory effect observed at a concentration of 50 µg/mL (Figure 1C and 1D). LPS stimulation was also found to upregulate the protein and mRNA expression of TLR4, p38, p-p38, p65, p-p65, and p-IκB in cells. In contrast, treatment with CGA at all three doses significantly downregulated the expression levels of these LPS-induced TLR4 pathway-related genes (Figure 1E and 1F). Notably, the inhibitory effects of CGA on these parameters were concentration-dependent.

### TLR4-mediated MAPK pathway regulates the inflammatory response of RAW264.7 cells

**Figure 2.**
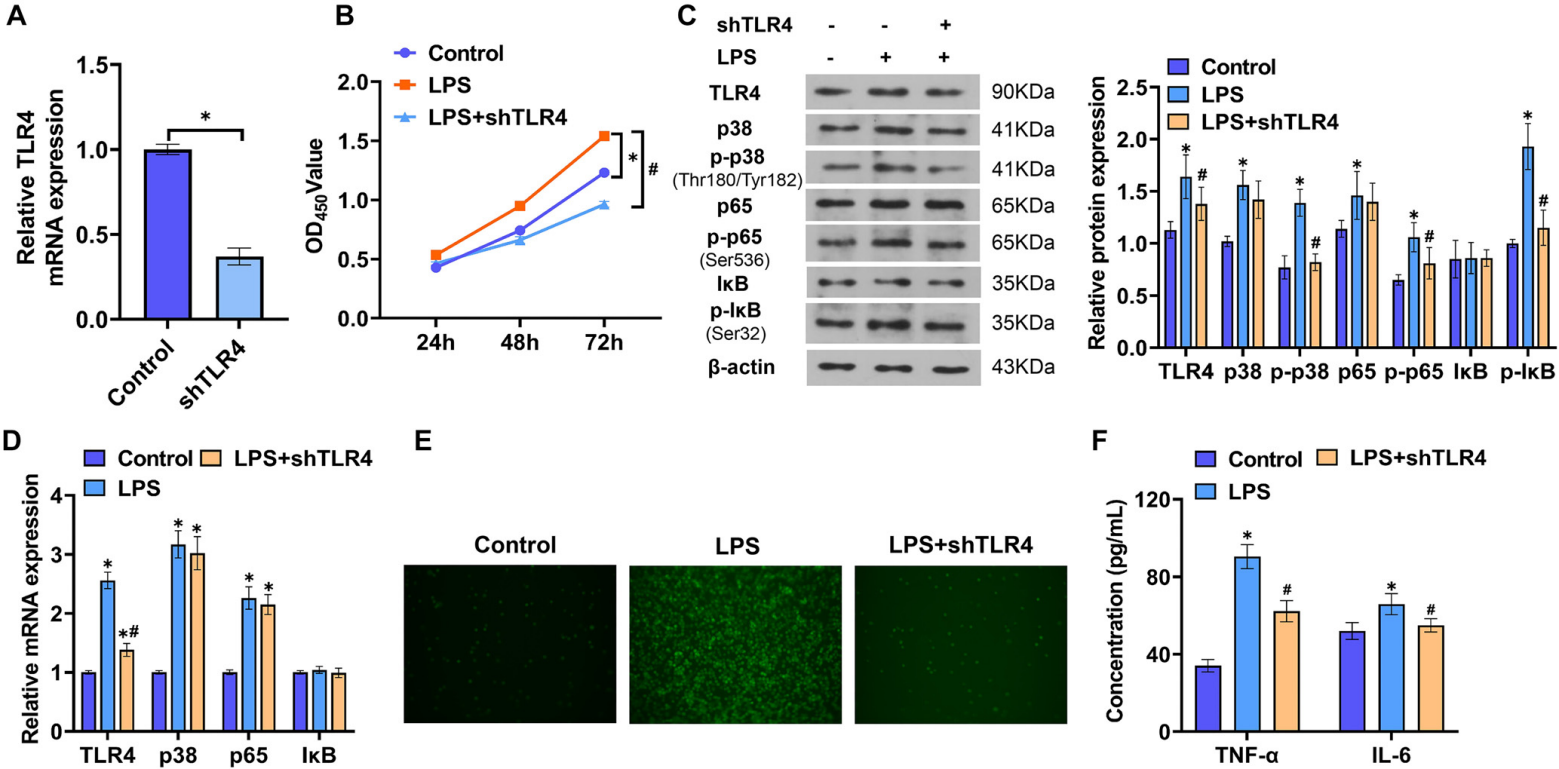
**Effect of downregulation of TLR4 expression on LPS-induced RAW264.7 cell function.** (A) Transfection efficiency of *shTlr4* was detected; (B) Effect of *shTlr4* transfection on cell proliferation was determined by CCK8 assay; (C) The protein expression of TLR4, p38, p-p38, p65, p-p65, IκB, and p-IκB were detected by western blotting; (D) The mRNA expression of TLR4, p38, p65, and IκB in cells was detected by qPCR; (E) The NO secretion in the cells was detected by immunofluorescence staining; (F) The secretion of TNF-α and IL-6 in cells was detected by ELISA. *N* ═ 3, *P < 0.05, compared with control group; ^#^*P* < 0.05, compared with LPS group. LPS: Lipopolysaccharide; TLR4: Toll-like receptor-4; shTLR4: Short hairpin RNA targeting TLR4; NO: Nitric oxide.

Based on the aforementioned studies, we designed a short hairpin RNA targeting TLR4 (shTLR4) to suppress TLR4 expression, which demonstrated high silencing efficiency in RAW264.7 cells (Figure 2A). Transfection with shTLR4 was found to inhibit the LPS-induced proliferative activity of these cells (Figure 2B). Protein assays further revealed that reduced TLR4 expression diminished the LPS-induced protein levels of TLR4, p-p38, p-p65, and p-IκB, as well as TLR4 mRNA expression (Figure 2C and 2D). Additionally, immunofluorescence staining and ELISA results indicated that downregulation of TLR4 expression effectively suppressed the LPS-induced secretion of inflammatory cytokines (Figure 2E and 2F).

**Figure 3.**
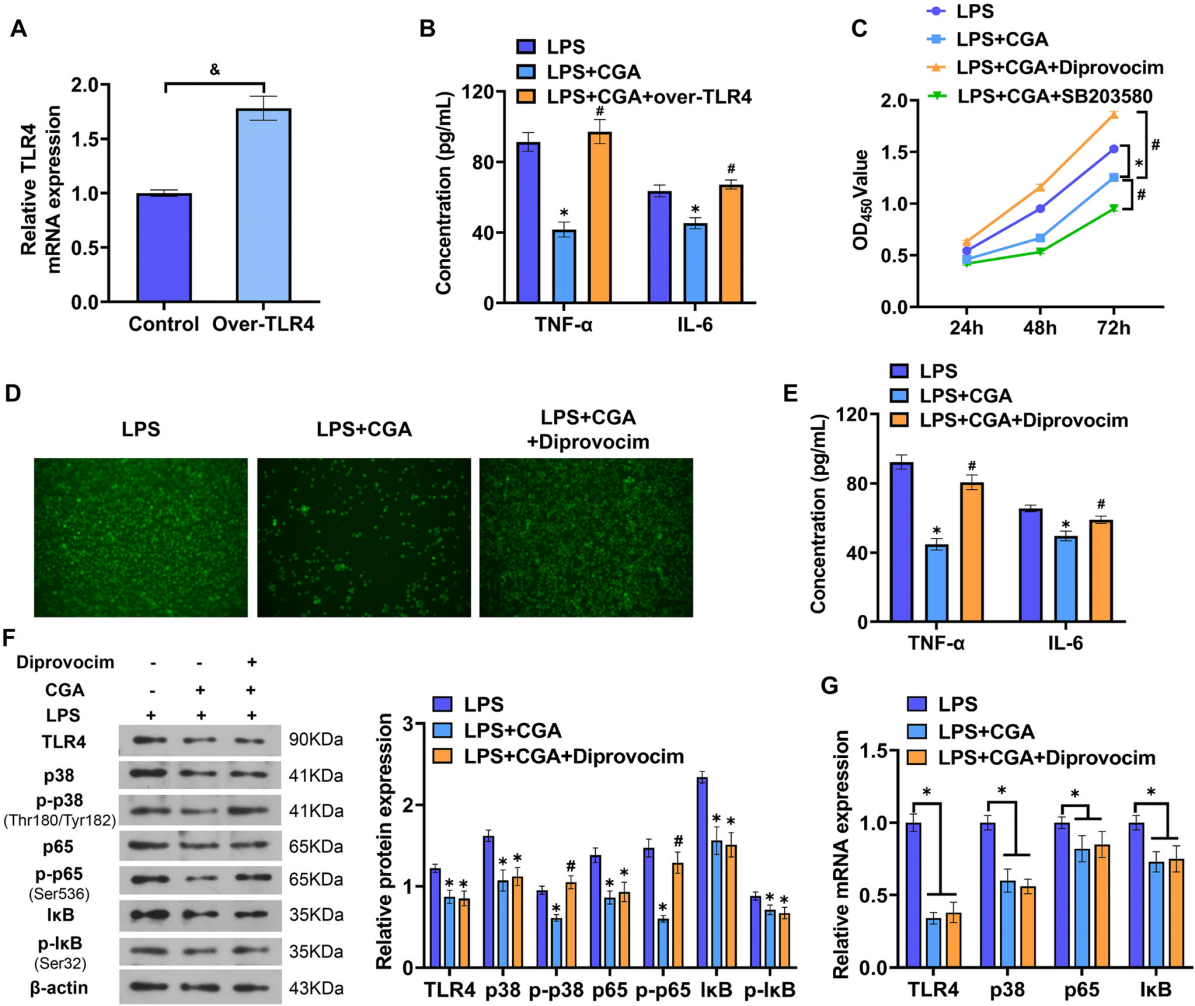
**Effect of TLR4/MAPK/NF-κB pathway activity on LPS-induced cell function.** (A) Over-TLR4 expression efficiency detection; (B) The effects of CGA and over-TLR4 transfection on the TNF-α and IL-6 levels in cells were detected by ELISA; (C) The changes of cell proliferative activity were detected by CCK8; (D) The NO secretion from the cells was detected by immunofluorescence staining; (E) The levels of TNF-α and IL-6 were determined by ELISA; (F) The protein expression of TLR4, p38, p-p38, p65, p-p65, IκB, and p-IκB was detected by western blotting; (G) The mRNA expression of TLR4, p38, p65, and IκB in cells was detected by qPCR. *N* ═ 3, ^&^*P* < 0.05, compared with control group; *P < 0.05, compared with the LPS group; ^#^*P* < 0.05, compared with the LPS+CGA group. LPS: Lipopolysaccharide; TLR4: Toll-like receptor-4; CGA: Chlorogenic acid; NO: Nitric oxide.

### Effects of CGA and TLR4/MAPK/NF-κB pathway on inflammatory response in RAW264.7 cells

We investigated the regulatory role of the TLR4/MAPK/NF-κB pathway in cellular inflammatory responses and constructed a TLR4 overexpression system (over-TLR4). PCR analysis revealed that over-TLR4 transfection significantly upregulated TLR4 mRNA expression (Figure 3A). Additionally, over-TLR4 transfection counteracted the anti-inflammatory effects of CGA, leading to increased TNF-α and IL-6 concentrations as well as enhanced cellular inflammatory activity (Figure 3B). To further explore these effects, LPS-activated cells were co-cultured with either an MAPK activator (1 nM), an MAPK inhibitor (SB203580, 5 µM), or a high dose of CGA (50 µg/mL). The MAPK inhibitor intensified the suppression of cell proliferation in the presence of CGA (Figure 3C). When cells were co-cultured with diprovocim and CGA, MAPK/NF-κB pathway activation was observed, which resulted in increased NO secretion, elevated TNF-α and IL-6 levels, and reduced the inhibitory effects of CGA compared to the LPS+CGA group (Figure 3D and 3E). Protein assays further demonstrated that diprovocim enhanced phosphorylation of p38, p65, and IκB (Figure 3F and 3G). In RAW264.7 cells, CGA inhibited the phosphorylation of TLR4 pathway proteins, an effect that was consistent with SB203580 treatment. These findings indicate that increased TLR4/MAPK/NF-κB pathway activity effectively reverses the anti-inflammatory effects of CGA on inflammatory cells.

### Effects of CGA on OVA-induced AR in mice

**Figure 4.**
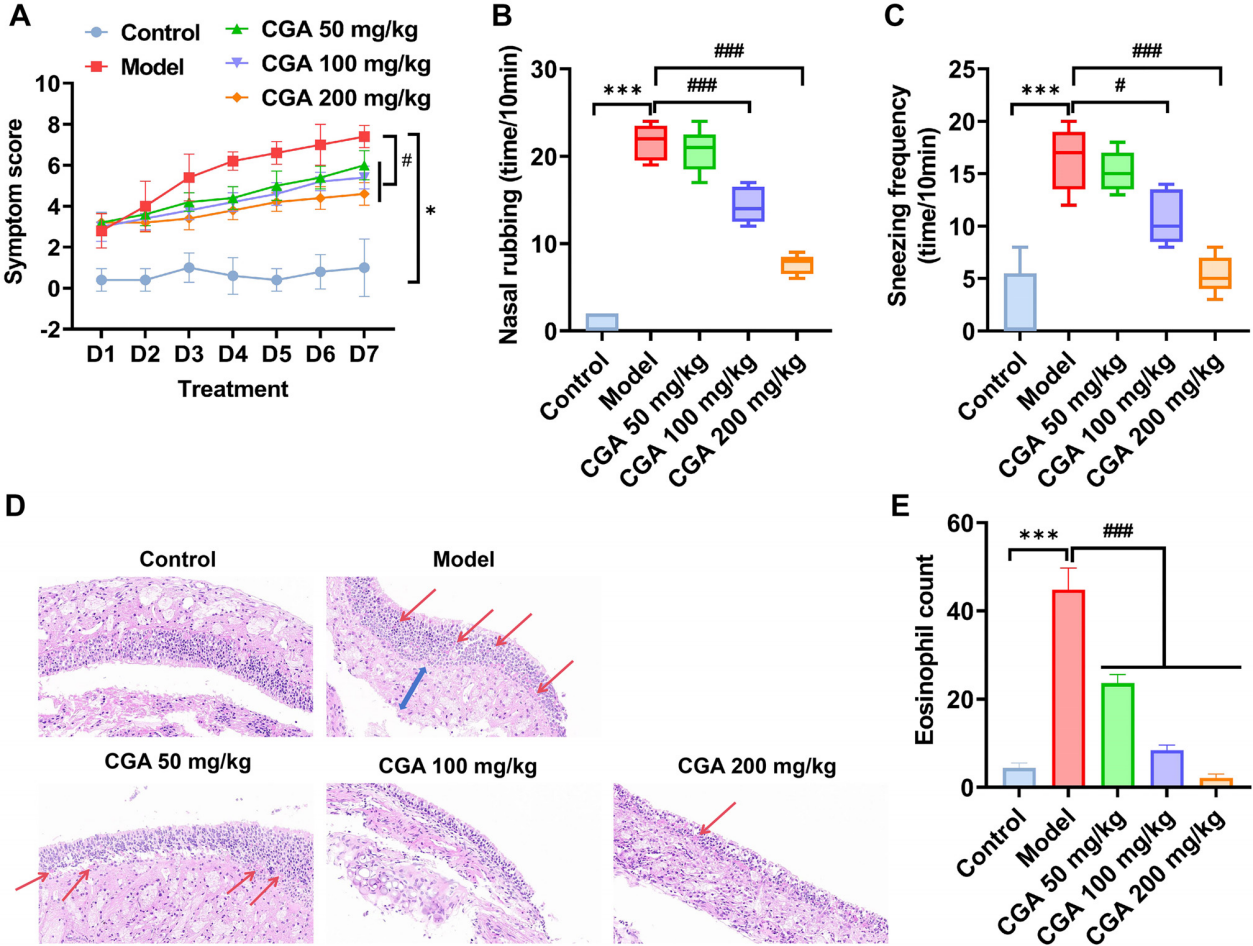
**Effects of different doses of CGA on mice with allergic rhinitis.** (A) Rhinitis behavioral score in mice; (B and C) The numbers of mice scratching their nose and sneezing after treatment; (D) Pathological staining analysis of nasal mucosa of mice (Red arrow points to eosinophils; blue arrow points to lamina propria of mucosa); (E) Analysis of blood eosinophil count in mice. *N* ═ 5, *P < 0.05, ***P < 0.001, compared with control group; ^#^*P* < 0.05, ^###^*P* < 0.001, compared with model group. CGA: Chlorogenic acid.

We established a mouse model of AR induced by OVA. Within seven days following local challenge with an OVA agonist, the behavioral score of AR mice increased significantly, indicating clear symptoms of rhinitis. This confirmed the successful establishment of the AR mouse model (Figure 4A). As shown in Figure 4B, after the final OVA challenge on day 27, the frequency of nose scratches and sneezes in AR model mice increased markedly. Treatment with three different doses of CGA alleviated these AR symptoms, with the group receiving 200 mg/kg CGA demonstrating the most pronounced therapeutic effect (Figure 4B and 4C). Histological analysis revealed significant inflammatory cell infiltration in the nasal mucosa of AR model mice, accompanied by congestion, swelling, and a marked increase in the thickness of the lamina propria. However, compared to untreated AR mice, CGA treatment significantly reduced mucosal inflammatory cell infiltration, with the high-dose group showing the most notable improvement (Figure 4D). Furthermore, CGA effectively decreased the number of eosinophils in the blood of AR mice (Figure 4E), supporting its potential therapeutic role.

### CGA alleviates inflammatory response through TLR4/MAPK/NF-κB pathway to treat AR in mice

**Figure 5.**
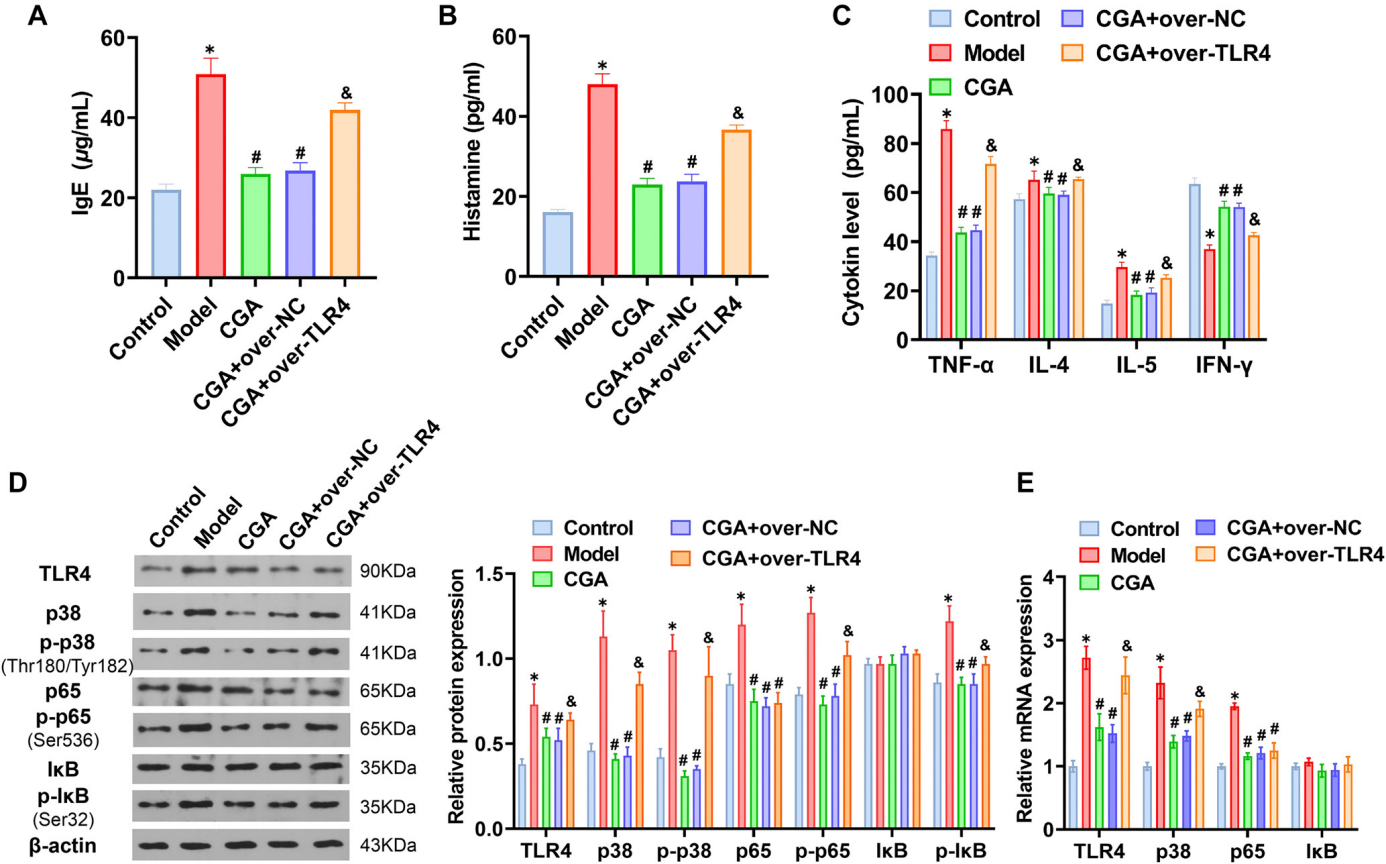
(A) Effect of CGA on inflammatory response and TLR4/MAPK/NF-κB pathway activity in mice with allergic rhinitis. (B) Determination of serum histamine and IgE levels in mice. Detection of serum IL-4, IL-5, IFN-γ, and TNF-α in mice. The protein (C) and mRNA (D) expression of TLR4, p38, p-p38, p65, p-p65, IκB, and p-IκB was detected by western blotting and qPCR. *N* ═ 5, *P < 0.05, ***P < 0.001, compared with control group; ^#^*P* < 0.05, ^###^*P* < 0.001, compared with LPS group; ^&^*P* < 0.05, compared with CGA+over-NC group. CGA: Chlorogenic acid; TLR4: Toll-like receptor-4.

When AR mice were treated simultaneously with CGA and over-TLR4, CGA treatment significantly reduced serum histamine and IgE levels. However, over-TLR4 transfection diminished CGA’s inhibitory effects on serum histamine and IgE levels (Figure 5A and 5B). Further analysis of serum inflammatory markers revealed that increased TLR4 expression reversed CGA’s inhibitory effects and enhanced the secretion of inflammatory factors (Figure 5C). Protein assays indicated that CGA mitigated OVA-induced increases in TLR4, p-p38, p-p65, and p-IκB protein expression, while over-TLR4 transfection elevated phosphorylation levels of components within the TLR4/MAPK/NF-κB signaling pathway (Figure 5D and 5E). These findings suggest that CGA suppresses OVA-induced inflammatory responses in mice by targeting the TLR4/MAPK/NF-κB pathway.

## Discussion

AR is not only a type I hypersensitivity reaction but also an inflammatory disease triggered by allergies. Inflammatory cells and their mediators exacerbate AR symptoms, contribute to recurrent episodes, and delay recovery [24]. Therefore, regulating inflammatory cells and mediators is crucial for the effective treatment of AR. The activation of various immune cells, such as macrophages, neutrophils, and lymphocytes, is a key driver of inflammatory reactions [25]. Studies have demonstrated that when patients with seasonal AR (SAR) are exposed to external pollen and treated with Artemisia annua extract SLIT, the polarization of M2-type macrophages significantly increases [26]. Macrophages can differentiate into the M1 type, leading to increased secretion of inflammatory factors, such as NO, IL-6, and TNF-α, which ultimately contribute to the progression of allergic diseases [27]. Mouse monocyte macrophage RAW264.7 cells, when stimulated by LPS, transform into the M1 type (pro-inflammatory), promoting inflammatory responses. These cells are frequently used as a cellular inflammation model for studying immune-related diseases [28]. Research has shown that CGA culture can significantly inhibit LPS-induced RAW264.7 cell activity and alleviate AR symptoms [18, 29]. This suggests that the therapeutic effects of CGA may involve the modulation of macrophage activity. In this study, a safe concentration of CGA in RAW264.7 cell cultures was determined to be 50 µg/mL, as higher concentrations inhibited cellular activity. A study by Park and Yoon [30] found that CGA exerts anti-inflammatory effects in LPS-PG-stimulated HGF-1 cells, with its effects increasing in a concentration-dependent manner and peaking at 50 µM. CGA was observed to inhibit the secretion of NO, TNF-α, and IL-6 in LPS-induced cells, demonstrating a significant anti-inflammatory effect. Related studies have also shown that CGA culture can markedly suppress LPS-induced inflammatory responses in RAW264.7 cells, reducing the secretion of inflammatory factors, such as IL-1β, IL-6, and TNF-α [31]. Furthermore, other research indicates that CGA can promote the transition of macrophages from the M2 phenotype to the M1 phenotype, aiding in tumor cell inhibition during cancer treatment [32, 33]. Taken together, the findings suggest that CGA culture in this study may inhibit inflammatory responses by promoting macrophage polarization toward the M2 type [34].

In addition, CGA inhibited the expression of TLR4, p-p38, p-p65, and p-IκB, consistent with findings from previous studies [35]. However, Cheng and Yeh [36] demonstrated that pretreatment with CGA enhanced LPS-induced expression of IL-10, IL-6, and NF-κB, suggesting an adaptive immunomodulatory effect. This highlights the need for further exploration of CGA’s regulatory effects on the inflammatory response in macrophages. Subsequent cell experiments revealed that, in an inflammatory *in vitro* cell model, transfection with shTLR4 in LPS-activated cells inhibited cellular activity, reduced phosphorylation of MAPK/NF-κB pathway components, and decreased the secretion of NO, TNF-α, and IL-6. Conversely, overexpression of TLR4 (via over-TLR4 transfection) counteracted CGA’s effects, increasing TNF-α and IL-6 concentrations and amplifying cellular inflammatory responses. Furthermore, activation of the MAPK/NF-κB pathway reversed CGA’s inhibitory effects, elevating inflammation-related factor expression. These results suggest that CGA suppresses macrophage inflammatory responses by inhibiting TLR4/MAPK/NF-κB pathway activation. Building on these *in vitro* findings, an *in vivo* AR animal model was developed to evaluate CGA’s therapeutic effects. Allergens commonly used to establish AR models include ovalbumin (OVA), 2,4-toluene diisocyanate (TDI), fungi, and pollen [37, 38]. Among these, OVA is a complete antigen known for its strong reproducibility and high animal survival rates. The AR model established with OVA closely resembles human AR in clinical symptoms and pathological changes, making it the most widely used model for AR research [38]. Observations, such as the number of sneezes, nasal scratches, and nasal secretion weights in AR animals provide a straightforward assessment of drug efficacy. Experimental results demonstrated that CGA effectively alleviated AR symptoms in mice, significantly reducing sneezing and nasal scratching while improving nasal mucosa edema and congestion. CGA also decreased the numbers of eosinophils and neutrophils, with its therapeutic effects showing a dose-dependent enhancement. Upon allergen entry, IgE antibodies are produced, which specifically bind to mast cells (MC) or basophils, triggering MC activation and the release of bioactive substances like histamine and kininogenase, thereby inducing AR [39]. CGA treatment reduced histamine and IgE levels, as well as the serum concentrations of inflammatory factors TNF-α, IL-4, and IL-6 in AR mice. However, increased TLR4 expression inhibited CGA’s anti-inflammatory effects.

Protein pathway analysis revealed that CGA treatment suppressed the activation of pathway proteins TLR4, p-p38, p-p65, and p-IκB. Additionally, TLR4 expression was found to inhibit the phosphorylation of pathway proteins p38, p65, and IκB. Thus, CGA appears to exert its anti-inflammatory effects through the TLR4/MAPK/NF-κB pathway, alleviating AR symptoms in mice. Compared to commonly used clinical therapies, such as second-generation antihistamines (e.g., loratadine, cetirizine), which rapidly relieve rhinitis symptoms caused by histamine release, CGA offers distinct advantages. Although antihistamines demonstrate significant efficacy, recent studies have highlighted their potential for severe cardiac toxicity and central nervous system side effects [40]. Nasal glucocorticoids, while effective for suppressing symptoms like nasal itching and sneezing, have been associated with adverse effects, including loss of smell, epistaxis, and headaches, as noted in long-term retrospective studies [41]. CGA showed significant therapeutic efficacy in AR mice without obvious toxic or side effects, suggesting it is a promising alternative. However, long-term administration of CGA may carry risks, as some studies suggest it could potentially induce cardiovascular diseases [42]. This study is limited by its small sample size and the lack of long-term efficacy observations. Thus, further research is needed to fully assess CGA’s safety and effectiveness for long-term use.

## Conclusion

Taking the findings together, this study demonstrated that CGA inhibited LPS-induced secretion of inflammatory cytokines by macrophages via the TLR4/MAPK/NF-κB pathway in a concentration-dependent manner. The AR mouse experiment further revealed that three doses of CGA alleviated the symptoms of OVA-induced AR to varying degrees, potentially through regulation of the same TLR4/MAPK/NF-κB pathway. However, this study did not analyze the role of macrophages in the development of AR in this mouse model. Future research could investigate macrophage activity in AR mice to determine whether the release of inflammatory factors triggered by macrophage activation contributes to the establishment of AR symptoms in mice.

## Data Availability

The data that support the findings of this study are available from the corresponding author upon reasonable request.
